# Cervical cancer screening and treatment, HIV infection, and age: Program implementation in seven regions of Namibia

**DOI:** 10.1371/journal.pone.0263920

**Published:** 2022-02-16

**Authors:** Abigail K. Korn, Laura Muzingwani, Gillian O’Bryan, Alison Ensminger, Ashley D. Boylan, Eva-Liisa Kafidi, Matheus Kashali, Laimi Ashipala, Anne-Marie Nitschke, Eric J. Dziuban, Norbert Forster, Linda O. Eckert, Gabrielle O’Malley

**Affiliations:** 1 International Training and Education Center for Health (I-TECH), Department of Global Health, University of Washington, Seattle, Washington, United States of America; 2 International Training and Education Center for Health (I-TECH), Department of Global Health, University of Washington, Windhoek, Namibia; 3 U.S. Centers for Disease Control and Prevention (CDC), Windhoek, Namibia; 4 Ministry of Health and Social Services—Namibia (MoHSS), Windhoek, Namibia; 5 Department of Obstetrics and Gynecology, University of Washington, Seattle, Washington, United States of America; Clinton Health Access Initiative, SOUTH AFRICA

## Abstract

The aim of this study was to assess differences in cervical cancer screening and treatment outcomes by HIV status in a routine programmatic setting with a high generalized HIV prevalence. Women living with HIV (WLHIV) are at heightened risk of developing cervical cancer and the World Health Organization recommends all WLHIV who are sexually active be screened, regardless of age. In 2018, Namibia’s Ministry of Health and Social Services introduced a screen-and-treat approach using visual inspection with acetic acid (VIA) and ablative treatment with cryotherapy or thermocoagulation with a focus on screening HIV-positive women due to Namibia’s 11.5% prevalence of HIV in women aged 15–49. Using program data from October 2018 to March 2020 from seven of the country’s 14 regions, we calculated descriptive statistics and chi-square tests to test the statistical significance of differences in VIA-positivity, ineligibility for ablative treatment, treatment completion, and same day treatment completion by HIV status. Between October 2018 and March 2020, the program conducted 14,786 cervical cancer screenings. Among 8,150 women who received their first VIA screening, more WLHIV screened VIA-positive (17%) than HIV-negative women (15%). This difference was statistically significant (p = 0.02). Among 2,272 women who screened VIA-positive at any screening, 1,159 (82%) completed ablative treatment. This suggests ablative treatment is feasible and acceptable in resource-limited settings. WLHIV were also more likely to complete treatment than HIV-negative women (p<0.01). Differences in health seeking behavior of sub-populations as well as resource availability between service delivery points should be considered for further investigation. Going forward in order to strengthen program implementation and expand screening access and uptake further investigation is needed to determine cancer incidence by HIV status, age, and time since last screening to assess cases that are averted as well as potential rates of overtreatment.

## Introduction

Cervical cancer is the most common cause of cancer-related deaths among women in low- and middle-Human Development Index (HDI) countries [1]. Its incidence varies considerably by region [1]. The southern Africa region has the second highest cervical cancer incidence rate in the world at 63.4 per 100,000 women, over five times the rates seen in North America and Western Europe [1]. In Namibia, data on cancer incidence in general, and on cervical cancer speifically, has been limited to date. The Global Cancer Observatory estimates that cervical cancer accounts for 20.5% of new cancer cases among females in Namibia [2].

Cervical cancer is highly preventable through vaccination against the types of human papilloma virus infection (HPV) most likely to cause cervical cancer, screening of all women, and treatment for precancerous lesions. Women living with HIV (WLHIV) are at heightened risk of developing cervical cancer, with data showing up to a six-fold increase in incidence compared to HIV-negative women [3]. Studies show both a higher prevalence and longer clearance time of HPV among WLHIV [3]. Thus, in countries such as Namibia which have high HIV prevalence and where the HPV vaccine is not yet widely available, screening and treatment of precancerous cells are essential for preventing cervical cancer.

The World Health Organization’s (WHO) cervical cancer elimination strategy has set ambitious targets for all countries to achieve by 2030: 90% of all girls vaccinated for HPV before age 15, 70% of all adult women screened by age 35 and again by age 45, and 90% of women identified with precancerous lesions treated [4]. Informed by the WHO 2013 guidelines, the 2018 Namibia policy is to provide cervical cancer screening and treatment for all women aged 30 or older in the general population [5]. For WLHIV, Namibia adopted the the WHO recommendation that all sexually active women be screened, regardless of age, due to their heightened risk [5]. Services are best offered through an integrated package that ensures a continuum of care in the provision of HIV and cervical cancer screening and treatment across the service delivery levels of the health system [6].

Beyond this general guidance, data were scant from randomized trials or programs in areas with a high burden of HIV that could further guide policy makers in refining their cervical cancer prevention approach. The 2021 WHO guidelines incorporate newer data into the recommendations but continue to cite low-certainty evidence for age to start screening for cervical cancer among WLHIV [7]. There is also little evidence on the outcomes of screening and completion of treatment for WLHIV compared to age-matched HIV-negative women [7]. In the context of limited randomized controlled trial data, it is useful to describe programmatic experience and outcomes of screening and treatment for cervical precancer to help inform policy decisions around resource allocation for optimum impact.

Namibia has a generalized HIV epidemic with an estimated prevalence of 11.5% among those aged 15–49 years: 14.8% among women and 8.0% among men [8]. The country’s 2018 population-based HIV impact assessment estimated that 89.5% of WLHIV knew their status, 97.1% of those were on treatment, and 92.2% of those on treatment were virally suppressed [8]. The age-standardized incidence of cervical cancer in Namibia is 24.2 per 100,000 [2]. In combination with a median age of sexual debut at 19 years among women, this context informed Namibia’s development of a population-based program for prevention of cervical cancer [9].

The Namibian Ministry of Health and Social Services (MoHSS) adopted cervical cancer prevention guidelines in March 2018 [10]. Although the guidelines include HPV vaccination, the vaccine is not yet available in the public sector and secondary prevention through screen-and-treat currently anchors the cervical cancer prevention program. Despite high awareness of cervical cancer, there is limited availability of cytology-based screening [11]. Pap smear coverage is 16% among family planning clients and coverage is constrained by limited cytology capacity in the national laboratory system which results in extended turnaround times for results [12]. HPV DNA testing is not yet available in public health facilities and Namibia’s screening relies on visual inspection with acetic acid (VIA), consistent with the WHO guidelines in place at the time when the Namibia policy was developed [5]. WHO guidelines specified that “where screening with an HPV test is not feasible, the panel suggests a strategy of screen with VIA and treat” [5]. Women who screen positive with VIA are offered same-day ablative treatment or referred for advanced treatment.

This study describes results from the first 18 months of a cervical cancer screening program which provided VIA as part of routine HIV services in seven of Namibia’s 14 regions (Erongo, Karas, Kavango East, Kavango West, Khomas, Oshikoto, and Otjozondjupa). These data are part of ongoing program monitoring and improvement as Namibia moves toward the WHO cervical cancer elimination goals. The analysis provides valuable insights into cervical cancer screening outcomes and treatment completion by HIV serostatus and age.

## Materials and methods

### Program implementation

The Namibia Cervical Cancer Screening and Treatment Programme launched in October 2018 with the training and certification of health care providers in VIA and cryotherapy and the establishment of those services in public health facilities in the Khomas region. The program was subsequently expanded to public health facilities in six additional regions, based on the volume of HIV-positive women active in care and treatment within the MoHSS cervical cancer screening target age range. The program also offered ablative treatment with cryotherapy and, beginning in September 2019, treatment with thermocoagulation.

### VIA eligibility

The MoHSS eligibility criteria for VIA included all women age 25–50 who were ever sexually active if HIV-negative or age 20–50 if HIV-positive. As part of routine service delivery, standard MoHSS written consent was documented for screening and treatment; no supplemental consent or contact with clients was sought for this retrospective analysis. Women who have had a hysterectomy with the cervix removed were ineligible for VIA screening. For HIV-positive women, VIA was recommended every three years or, if a woman screened VIA-positive and received treatment, every year until she screened VIA-negative for three consecutive years. For HIV-negative women, VIA was recommended every five years or, if she screened VIA-positive and received treatment, every year until she screened VIA-negative for three consecutive years. Women of unknown HIV status followed the HIV-positive screening guidelines.

### Treatment eligibility

Women screening VIA-positive were eligible for cryotherapy if the lesions occupied less than 75% of the transformation zone, the entire lesion was visible, the lesion did not extend into the cervical os and the cryotip could completely cover the lesion. Ineligibility criteria included visible abnormal blood vessels, presence of polyps or scarring that prevented full contact between the cryotip and cervix, pregnancy, and severe cervicitis. Eligibility criteria for thermocoagulation were similar to cryotherapy except that the thermocoagulation tip does not need to cover the lesion completely. If screening results indicated treatment, the client could elect same-day treatment or delayed treatment, as needed. If the lesions did not meet the above criteria, the clients were referred for large loop excision of the transformation zone (LLETZ) which required a return visit based on the availability of a trained provider at centrally located sites. Clients with suspected cancer were not considered VIA-positive and were referred for treatment.

### Data collection

De-identified data were abstracted from paper-based health facility cervical cancer screening registers into Excel. These registers are the primary data collection and reporting tool used by MoHSS for cervical cancer screening. Data quality checks are performed monthly to identify outlying data and changing trends, verify data, and rectify register data if needed. Variables of interest were age, HIV status, screening type, VIA outcome, and treatment outcome. Screening type included first screening, re-screening, and one-year post-treatment follow-up screening. Data on cytology-based screening and treatment outcomes were not captured in the register. Treatment outcomes for suspected cancer cases were not captured at the referring health facility and the de-identified data used for this study prevented stable record matching of biopsy results at the referral facility.

### Data analysis

We calculated descriptive statistics using Stata, version 15, and used chi-square tests to test the statistical significance of differences by HIV status and VIA-positivity, ineligibility for ablative treatment, treatment completion, and same day treatment completion using an alpha of 0.05. The study obtained all required approvals from the Office of the Executive Director of the Namibian MoHSS and received a non-research determination from the University of Washington Institutional Review Board. The study was reviewed in accordance with the U.S. Centers for Disease Control and Prevention human research protection procedures by the Office of the Associate Director for Science and was determined to be a non-research, public health program activity. The information included in this cross-sectional analysis was routinely collected, programmatic data. Consent was not obtained since the data used for analysis were anonymized.

## Results

Between October 2018 and March 2020, a total of 121 healthcare workers (doctors, registered and enrolled nurses) were trained in VIA and ablative treatment. Ten doctors were also trained to perform LLETZ and cervical biopsies starting in February 2020. Four nurse mentors also provided supportive supervision including review of cervicograms, as well as monitoring the WHO program performance benchmark indicators as part of routine quality assurance. During its first 18 months, the Namibian screen-and-treat program conducted a total of 14,786 screenings, including 8,150 first screenings, and provided 1,804 instances of treatment with ablative treatment or LLETZ at 50 public health facilities in the above stated regions.

Among 8,150 women receiving their first VIA screening, 4,805 (59%) were HIV-positive, 3,202 (39%) were HIV-negative, and 143 (2%) had unknown HIV status (see Table 1). Among WLHIV, 802 screened VIA-positive (17%) compared to 475 among HIV-negative women (15%). This difference was statistically significant when using a chi-square test (p = 0.02).

**Table 1:** VIA findings at first screening by age, lesion severity, and HIV status in Namibia (October 2018–March 2020).

Age (years)										
	Total	15–19	20–24	25–29	30–34	35–39	40–44	45–49	≥50	Unknown
**HIV-positive**										
VIA-screened	4,805	5	339	658	906	1,077	1,025	770	22	3
VIA-positive*	802 (17%)	0 (0%)	80 (24%)	131 (20%)	167 (18%)	198 (18%)	151 (15%)	73 (9%)	1 (5%)	1 (33%)
Eligible for ablation^†^	522 (65%)	0 (0%)	57 (71%)	88 (67%)	99 (59%)	134 (68%)	100 (66%)	42 (58%)	1 (100%)	1 (100%)
Ineligible for ablation^†^	280 (35%)	0 (0%)	23 (29%)	43 (33%)	68 (41%)	64 (32%)	51 (34%)	31 (42%)	0 (0%)	0 (0%)
Suspected cancer*	46 (1%)	0 (0%)	4 (1%)	5 (1%)	5 (1%)	6 (1%)	13 (1%)	11 (1%)	2 (9%)	0 (0%)
**HIV-negative**										
VIA-screened	3,202	9	282	1,150	676	481	343	229	26	6
VIA-positive*	475 (15%)	2 (22%)	51 (18%)	186 (16%)	114 (17%)	71 (15%)	29 (8%)	20 (9%)	1 (4%)	1 (17%)
Eligible for ablation^†^	326 (69%)	2 (100%)	32 (63%)	137 (74%)	71 (62%)	47 (66%)	22 (76%)	14 (70%)	1 (100%)	0 (0%)
Ineligible for ablation^†^	149 (31%)	0 (0%)	19 (37%)	49 (26%)	43 (38%)	24 (34%)	7 (24%)	6 (30%)	0 (0%)	1 (100%)
Suspected cancer*	17 (1%)	0 (0%)	2 (1%)	2 (0%)	4 (1%)	2 (0%)	2 (1%)	3 (1%)	2 (8%)	0 (0%)
**HIV Unknown**										
VIA-screened	143	0	15	47	31	21	13	11	3	2
VIA-positive*	31 (22%)	0 (0%)	3 (20%)	13 (28%)	6 (19%)	7 (33%)	2 (15%)	0 (0%)	0 (0%)	0 (0%)
Eligible for ablation^†^	23 (74%)	0 (0%)	3 (100%)	11 (85%)	4 (67%)	4 (57%)	1 (50%)	0 (0%)	0 (0%)	0 (0%)
Ineligible for ablation^†^	8 (26%)	0 (0%)	0 (0%)	2 (15%)	2 (33%)	3 (43%)	1 (50%)	0 (0%)	0 (0%)	0 (0%)
Suspected cancer*	2 (1%)	0 (0%)	0 (0%)	0 (0%)	0 (0%)	0 (0%)	1 (8%)	0 (0%)	1 (33%)	0 (0%)

* Among VIA-screened.

† Among VIA-positive.

Of the 1,308 VIA-positive women receiving their first screening, 437 (33%) were ineligible for cryotherapy or thermocoagulation treatment. Ineligibility for ablative treatment was more common among WLHIV (35%) compared to HIV-negative women (31%) at first screening although this difference was not statistically significant (p = 0.36). WLHIV were more likely to present with suspected cancer (16%) compared to HIV-negative women (11%) at first screening.

Among 2,272 women who screened VIA-positive, including first screening, follow up, and rescreening, 1,159 (82%) completed ablative treatment. Treatment completion was higher among WLHIV (86%) compared to HIV-negative women (77%, see Table 2). This difference was statistically significant (p<0.01) using a chi-square test. Treatment completion rates were lower among 25-30-year-olds by 8% among WLHIV and by 5% among HIV-negative women compared to the 20-24-year-olds. There was a slight increase in treatment completion rates among women aged 40 years and older. VIA-positive WLHIV completed treatment on the same day more often than HIV-negative women (68% vs. 56%, see Table 3). This difference was statistically significant (p = 0.01) and held across age groups except for women aged 50 years or older, where both WLHIV and HIV-negative women received same day treatment in 67% of cases. Among 804 women referred for LLETZ after any screening, 621 (77%) completed treatment.

**Table 2:** Treatment for cervical pre-cancer lesions and post-treatment complications by age, lesion severity, and HIV status in Namibia (October 2018–March 2020).

		Age (years)								
	Total	15–19	20–24	25–29	30–34	35–39	40–44	45–49	≥50	Unknown
**HIV-positive**										
VIA-positive	1,433	0	93	181	246	379	337	187	8	2
Eligible for ablation*	855 (60%)	0 (0%)	64 (69%)	112 (62%)	144 (59%)	244 (64%)	189 (56%)	98 (52%)	3 (38%)	1 (50%)
Ablation performed^†^	733 (86%)	0 (0%)	58 (91%)	93 (83%)	121 (84%)	210 (86%)	163 (86%)	84 (86%)	3 (100%)	1 (50%)
Post-treatment complication	6	0	1	0	2	1	0	1	0	1
**HIV-negative**										
VIA-positive	839	2	67	282	223	129	74	56	5	1
Eligible for ablation*	553 (67%)	2 (100%)	44 (67%)	197 (68%)	140 (63%)	81 (63%)	51 (69%)	35 (63%)	3 (60%)	0 (0%)
Ablation performed^†^	426 (77%)	1 (50%)	36 (82%)	152 (77%)	106 (76%)	61 (75%)	40 (78%)	27 (77%)	3 (100%)	0 (0%)
Post-treatment complication	2	0	0	1	1	0	0	0	0	0

* Among VIA-positive women.

† Among women eligible for ablation.

**Table 3:** Same day cryotherapy/thermocoagulation performed by HIV status in Namibia (October 2018 –March 2020).

		Age (years)								
	Total	15–19	20–24	25–29	30–34	35–39	40–44	45–49	≥50	Unknown
**HIV-positive**										
VIA-positive	1,433	0	93	181	246	379	337	187	8	2
Ablation eligible	855	0	64	112	144	244	189	98	3	1
Ablation performed, same day	583 (68%)	0 (0%)	46 (72%)	78 (70%)	93 (67%)	168 (69%)	125 (66%)	71 (72%)	2 (67%)	0 (0%)
Ablation performed, delayed	150	0	12	15	28	42	38	13	1	1
**HIV-negative**										
VIA-positive	839	2	67	282	223	129	74	56	5	1
Ablation eligible	553	2	44	197	140	81	51	35	3	0
Ablation performed, same day	311 (56%)	0 (0%)	28 (64%)	100 (51%)	76 (54%)	52 (64%)	32 (63%)	21 (60%)	2 (67%)	0 (0%)
Ablation performed, delayed	115	1	8	52	30	9	8	6	1	0

Complications following ablative treatment occurred in 6 of 733 (0.8%) WLHIV compared to 2 of 426 (0.5%) HIV-negative women (see Table 2).

## Discussion

Namibia’s cervical cancer screening and treatment program provided 14,786 VIA screenings in the selected regions over the first 18 months. We found significantly higher rates of VIA-positivity, treatment completion, and ineligibility for ablative treatment among WLHIV when compared with HIV-negative women.

The significantly higher rates of VIA positivity among WLHIV in our study (17% vs 15%) align with other published literature, although the difference is not as large as seen in some African programs. Results ranged from 40% vs 20% in Zambia, 14% vs 5% in South Africa, 12% vs 3% in Tanzania and 8% vs 2% in Cote d’Ivoire [13–16]. The similarity in VIA positivity rates in Namibia may indicate that the WLHIV in our sample were on antiretroviral therapy and virally suppressed; findings from NAMPHIA reported that 92% of WLHIV in Namibia are virally suppressed. A 2017 systematic review proposed that viral suppression through ART supports the immunological response to prevent and/or clear HPV, though this merits further investigation [17]. WLHIV aged 20–24 had the highest VIA positivity rate. This is consistent with the epidemiology of HPV infections which are more common in women that are younger, and then resolve at a later stage.

Our findings support the integration of cervical cancer screening programs into HIV services and align with the Sigfrid et al. systematic review that found integration was feasible and acceptable to WLHIV [18]. However, the VIA positivity rate of 15% among HIV-negative women suggests that all women in this population benefit from cervical cancer screening and treatment. This is double the cytology-diagnosed prevalence of 7.7% for cervical intraepithelial neoplasia or more advanced cervical disease previously reported in a sample of 684 Namibian women [19]. However, the VIA positivity rate falls within the range of 8–28% VIA-positivity found in other sub-Saharan African program implementation settings [13,20–23]. Furthermore, this rate meets the WHO benchmark for program quality monitoring of 5–25% VIA positivity in a previously unscreened general population [24]. A known limitation of VIA screening is an increase in false-positive results among populations with elevated rates of cervicitis and further studies are needed to understand the potential role cervicitis may play in VIA screening in Namibia [25].

Namibia’s treatment completion rates suggest that ablative treatment is feasible and acceptable in resource-limited settings. Logistical challenges for providing cryotherapy, such as availability of the compressed gas refill tanks, may have negatively impacted treatment completion rates and time to treatment when only cryotherapy was available. Programs in Zambia and Malawi found similar overall treatment completion rates when using cryotherapy and thermal ablation, respectively [26,27]. However, in contrast to results from Zambia where treatment completion was similar across HIV serostatuses [13], we observed a significantly higher treatment completion rate among WLHIV (86% vs. 77%) in Namibia. Although screening and treatment services were available to women regardless of HIV status, there were sites where services were offered in HIV care centers or where limited access to necessary equipment may have impacted results. Differences in health seeking behavior between WLHIV and HIV-negative women, as well as differences in resource availability between HIV services and integrated primary health care models, such as staffing volume and supplies, should be considered further as factors that could influence observed differences.

The post-ablative treatment complication rate in our routine service settings was low. There is limited published data on adverse events following ablative treatment, however the published literature suggests complications are rare [28].

Ineligibility for ablative treatment serves as a proxy for size and severity of the lesion. Among all VIA-positive women receiving their first screening, 437 (33%) were ineligible for ablative treatment, higher than the 21% ineligibility rate found in Nigeria [29]. In our study, HIV-positive women were more likely to be ineligible for treatment (35%) compared to HIV-negative women (31%). These results highlight the importance of availability and linkage to advanced treatment options in settings with a high HIV burden. A LLETZ treatment completion rate of 77% is promising and continues to be expanded in Namibia’s program. Data on LLETZ treatment completion by HIV status were not available.

The Namibia policy to start cervical cancer screening at age 20 for WLHIV seems appropriate. Among 20-24-year-old women, more HIV-positive women were VIA-positive than HIV-negative women (24% and 18%, respectively). However, it is unclear how many of these lesions would have self-cleared with time, especially among younger women, and how many would have progressed to cervical cancer. Of the 65 cases of suspected cancer at first screenings, 4 cases were among WLHIV aged 20–24. More information on the pathology of these cases and ongoing monitoring of cancer incidence by age, HIV status, and last screening date is needed to guide policymakers in refining screening policies, especially the age at which to begin screening and the impact of VIA on reducing cancer incidence. Further research on knowledge, attitudes, and practices around HPV and cervical cancer screening and treatment in Namibia could strengthen awareness campaigns and counseling strategies.

### Limitations

This study used aggregate, observational program data. Data quality checks performed indicate that the data are reliable, and efforts were made to cross-check data with clinical records. However, it is not possible to discern the number of unique women screened or treated; rather, the number of screenings and treatments conducted is reported. Similarly, it is not possible to confirm whether the same women who screened positive were also those who were treated in the reporting period or to track one-year post-treatment follow-up visits.

## Conclusions

The Namibia Cervical Cancer Screening and Treatment Programme successfully provided, as part of routine HIV service delivery, 14,786 screenings in the first 18 months of implementation. The program continues to expand, supporting Namibia’s progress towards the WHO cervical cancer elimination targets. These results support the use of VIA programs in high HIV burden settings until widespread HPV testing to triage clients is feasible. Further research into the implementation considerations for incorporating HPV tests into screening and launching an HPV vaccination program might also support policymakers. Further investigation is needed into cancer incidence by HIV status, age, and time since screening to estimate potential rates of overtreatment and cancer cases averted in young women as well as ongoing analyses such as return rates for one-year post-treatment follow-ups and VIA-positivity following treatment.

## Supporting information

S1 File Inclusivity in global research questionnaire. (DOCX)
